# The epigenetic implication in coronavirus infection and therapy

**DOI:** 10.1186/s13148-020-00946-x

**Published:** 2020-10-21

**Authors:** Sandra Atlante, Alessia Mongelli, Veronica Barbi, Fabio Martelli, Antonella Farsetti, Carlo Gaetano

**Affiliations:** 1 Laboratorio di Epigenetica, Istituti Clinici Scientifici Maugeri IRCCS, Via Maugeri 4, 27100 Pavia, Italy; 2 Laboratorio di Cardiologia Molecolare, Policlinico San Donato IRCCS, Milan, Italy; 3 Institute for Systems Analysis and Computer Science “A. Ruberti” (IASI), National Research Council (CNR), Rome, Italy

**Keywords:** Coronaviruses, Epigenetics, Nucleic acids, Aging, Metabolism, Chronic disease

## Abstract

Epigenetics is a relatively new field of science that studies the genetic and non-genetic aspects related to heritable phenotypic changes, frequently caused by environmental and metabolic factors. In the host, the epigenetic machinery can regulate gene expression through a series of reversible epigenetic modifications, such as histone methylation and acetylation, DNA/RNA methylation, chromatin remodeling, and non-coding RNAs. The coronavirus disease 19 (COVID-19) is a highly transmittable and pathogenic viral infection. The Severe Acute Respiratory Syndrome Coronavirus 2 (SARS-CoV-2), which emerged in Wuhan, China, and spread worldwide, causes it. COVID-19 severity and consequences largely depend on patient age and health status. In this review, we will summarize and comparatively analyze how viruses regulate the host epigenome. Mainly, we will be focusing on highly pathogenic respiratory RNA virus infections such as coronaviruses. In this context, epigenetic alterations might play an essential role in the onset of coronavirus disease complications. Although many therapeutic approaches are under study, more research is urgently needed to identify effective vaccine or safer chemotherapeutic drugs, including epigenetic drugs, to cope with this viral outbreak and to develop pre- and post-exposure prophylaxis against COVID-19.

## Introduction

The severe acute respiratory syndrome coronavirus 2 (SARS‐CoV‐2) is a severe new disease that emerged in Wuhan, China, in December 2019 [1]. The World Health Organization (WHO) has acknowledged the SARS-CoV-2/coronavirus disease 2019 (COVID-19) epidemic as a public health emergency of international concern [2]. The WHO reported that, until this manuscript submission on September 26, 2020, there had been 32,344,734 confirmed cases and 984,902 deaths worldwide [3]. Zhou and coworkers identified this novel coronavirus using next-generation sequencing of nucleic acids in the broncho-alveolar lavage fluids of diseased patients [1]. The name SARS-CoV-2 was assigned since its RNA virus genome is closely related to the severe acute respiratory syndrome (SARS)-CoV, which emerged in human species in 2003–2004, and the infection is associated with a SARS-like disease [4, 5]. The cross-species transmission of SARS-related CoVs, causing virulent pandemic infections, represents a significant threat to the human population. This episode is the third CoV epidemic after SARS (2003) and the Middle East respiratory syndrome (MERS-2012) CoV outbreaks. Initial phylogenetic analysis indicated the formation of a common cluster with bat SARS-like CoV isolated in 2015 [6], and structural studies showed the close relation of the receptor‐binding domain of SARS‐CoV‐2 with the other CoV [7]. COVID-19 symptoms include fever, cough, shortness of breath, fatigue, and gastrointestinal distress, and the disease can aggravate, leading to severe pneumonia, severe symptomatic acute respiratory distress syndrome (ARDS), entailing several issues such as cardiovascular complications, kidney injury, stroke, and mortality [8, 9]. Of note, among patients infected by SARS-CoV-2, adults over 65 years of age represent 80% of hospitalizations and have a 23-fold higher risk of death than those under 65 [10]. The aging process leads to changes at the cell, tissue, and organ levels, known as hallmarks of aging, that contribute to morbidity, frailty, and mortality in the elderly [11]. These aging hallmarks affect many cellular and system functions and play relevant roles in several chronic diseases. Hence, they might influence viral infections. Some of these hallmarks include inflammation, adaptive immunosenescence, genomic instability, mitochondrial dysfunction, and epigenetic alterations [11]. Since COVID-19 mortality factors are similar to those of SARS and MERS, when treating COVID-19 infected older patients, the aging hallmarks should be critically considered to improve any positive outcome [12–14].

## Coronaviruses

**Fig. 1:**
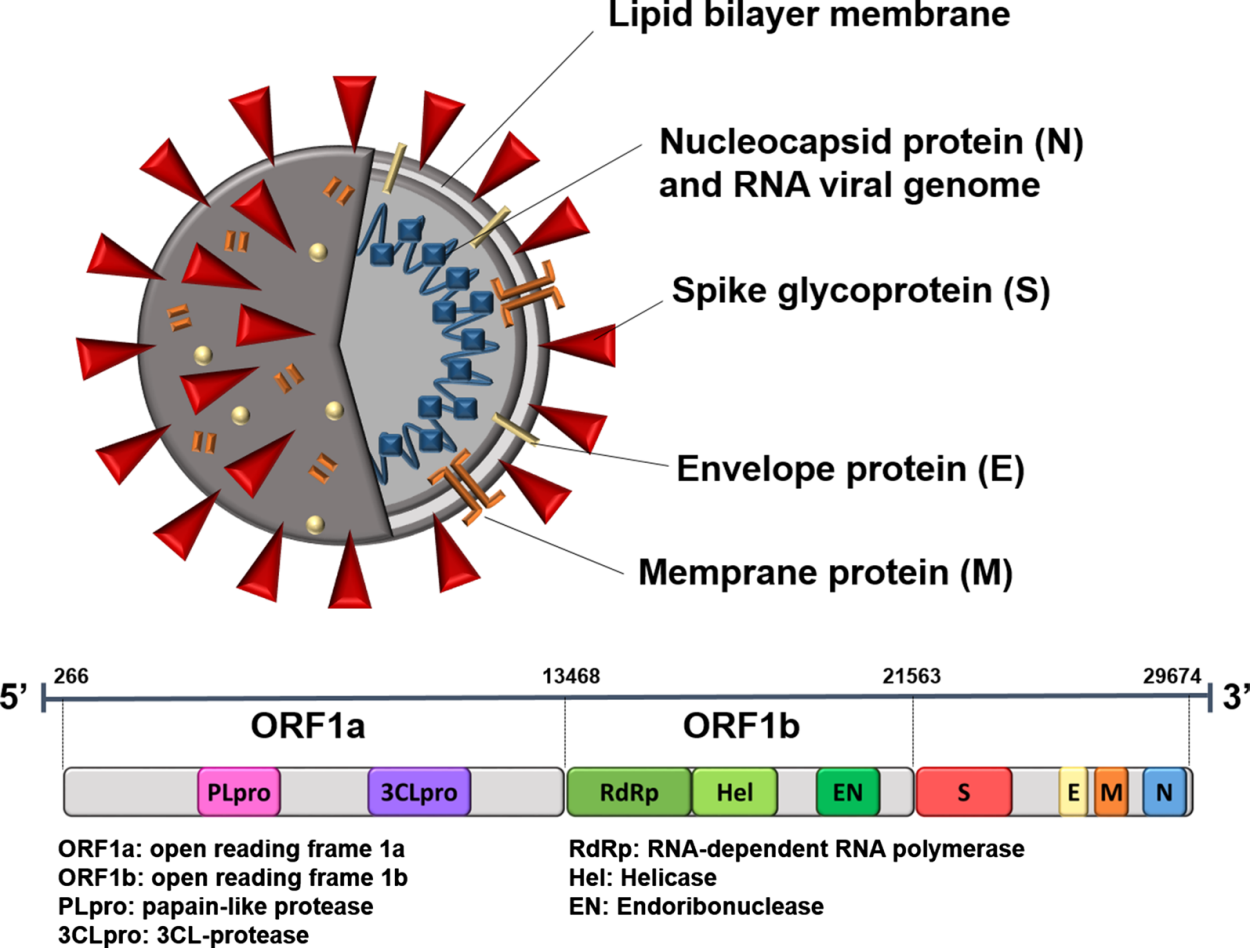
Schematic representation of SARS-CoV-2 structure. The single-stranded RNA viral genomic assembly of 29,674 nucleotide base pair encodes open reading frame 1a, open reading frame 1b, Spike glycoprotein, Envelope, Membrane, and Nucleocapsid proteins. ORF1a gene encodes papain-like protease and 3CL protease, ORF1b gene encodes an RNA-dependent RNA polymerase, a helicase, and an endoribonuclease

Coronaviruses belong to the *Coronaviridae* family in the Nidovirales order. They are small in size (65–125 nm in diameter) and contain a single-stranded RNA as a nucleic material, from 26 to 32 kb long. The subgroups of the coronaviruses family are alpha (α), beta (β), gamma (γ), and delta (δ) coronavirus [15]. Sars-CoV-2 belongs to the β-coronavirus genus [16]. CoVs are enveloped positive‐stranded RNA viruses owning a relatively large genome of approximately 30 kb, organized in 10 open‐reading frames (ORFs; Fig. 1). The 5′ region of the viral genome encodes for ORF1a and ORF1b. They are translated in the 1a and 1b polyproteins. These polypeptides are cleaved into a set of nonstructural proteins (Nsp) by viral and cellular proteases. Some amino acids are encoded by one codon, while several alternative codons, known as synonymous codons, encode other viral polypeptides, affecting translation efficiency, which differs from one organism to another [17, 18]. Specifically, coronaviruses comprise four structural proteins, namely spike (S), nucleocapsid (N) envelope (E), and membrane (M) [17]. The spike protein is a glycoprotein responsible for virus attachment to the receptor and fusion with the cell membrane, through the S1 receptor-binding domain and the S2 subunit, respectively [19]. The N protein is involved in genome replication and interacts with the viral RNA to form the ribonucleoprotein [20]. The E protein helps in virions assembly and comprises ion channel actions [21], and the M protein is necessary for the assembly of new virus particles [22]. The most threatening bat-derived CoVs are those with distinctively human-tropic S proteins. Inside the human lungs, the S protein interacts with several host susceptibility factors, such as receptors and proteases, leading to a substantial protein conformational arrangement, which allows the virus-cell membrane fusion and, therefore, the infection [4]. Specifically, using the isolated SARS-CoV-2 virus, Zhou et al*.* demonstrated that SARS-CoV-2 spikes bind human Angiotensin-converting enzyme 2 (ACE2) receptor [23], a cell surface receptor that converts the vasopressor octapeptide angiotensin-II to the vasodilator angiotensin1-7 (Ang1-7). ACE2 is highly expressed in the vascular endothelia, lung, kidney, small intestine epithelial cells, immune cells, and testis [24, 25]. Following binding to ACE2, the virus enters the cell either through an endosome or by S glycoprotein cleavage. This process involves host cell proteases, such as TMPRSS2 and furin. Inside the host cell, the CoVs genome is replicated in the cytoplasm, using the viral RNA polymerase, while the host ribosome machinery is used for protein synthesis. Then, in the host endoplasmic reticulum-Golgi intermediate complex (ERGIC), the virion is finally assembled, and the mature viruses are incorporated in small smooth-walled vesicles and secreted by the host cells [26]. These mechanisms were already observed in the earlier SARS-CoV. Of note, also SARS-CoV (2003) employs the host ACE2 protein as the principal receptor and shows a zoonotic distribution, according to the ACE2 receptor orthologues [27]. A similar process is observed during MERS-CoV infections, but the virus binds to a different protein receptor, the dipeptidyl peptidase 4 (DPP4), or its orthologues in the other animal species [28]. Noteworthy, DPP4, also known as CD26, is a surface protein recognized by natural killer (NK) cells, particularly abundant in senescent cells [29]. As a protease, DPP4 can inactivate incretins, triggering a rapid release of insulin from pancreatic β cells, affecting glucose homeostasis, which has particular relevance in the aging population [29]. Recently, Vankadari et al*.*, taking advantage of a docked complex model of the SARS-CoV-2 spike glycoprotein and DPP4, showing a large interface between the proteins, suggested that the human DPP4/CD26 may interact with the S1 domain of the viral spike glycoprotein [30]. This structural feature indicates additional possibilities of virus–host interaction other than ACE2 and suggests a close similarity with other coronaviruses that use DPP4 as a functional receptor [31].

## Epigenetics

Epigenetics has been described as the area of life sciences that studies stably heritable phenotype resulting from changes in chromatin structural/activation states without altering the DNA primary nucleotide sequence [32]. Currently, the epigenomics—the study into genome epigenetics—gives us the capability to read, locate, and interpret functionally the epigenetic machinery that controls the entire genome at various levels [31]. During the last decades, a large body of research provided evidence that epigenetics plays an important role in the establishment and progression of many common diseases, particularly those related to age (age-related diseases; ARD) [33]. Moreover, the patterns of gene expression determined by molecular epigenetic marks established during development affect the vulnerability to several diseases in humans, including viral infections [18]. Remarkably, epigenetic alterations involve changes in the chromatin structure or the nucleic acid chemical properties without hacking the genetic code, unlike mutations that directly derange the genetic material [32]. This characteristic makes epigenetic alterations reversible, flexible, and quickly responsive to environmental changes and other exposures [34]. Indeed, prolonged exposure to altered metabolic conditions may epigenetically affect human cells [35]. Several epigenetic mechanisms work together to regulate gene expression synchronizing the metabolic information. At the chromatin level, DNA methylation and histone modifications lead to chromatin remodeling, and together with other modifying proteins (sirtuins, prions, etc.) and non-coding RNA (miRNA, sRNA, lncRNA), allow chromatin access to proteins that regulate DNA transcription and, therefore, RNA and protein synthesis [36, 37]. The cell epigenome reflects the gene activation state of chromatin by encoding the information about how and where gene-specific activation switches are located and used in the genome [38]. Chromatin is a complex of proteins and DNA; the nucleosome, composed of two copies of four core histones (H3, H4, H2A, and H2B), is the fundamental unit. The DNA wraps around the histone octamer, which, thanks to histones specific chemical composition, regulates DNA access for gene transcription [39]. Chromatin remodeling controls many epigenetic processes through a series of dynamic changes in the structural organization of nucleosome by reversible histone and DNA modifications, which result in different chromatin condensation levels [40]. Among the hundreds of enzymes involved in the epigenetic regulation of relevance for this article are histone acetyltransferases (HATs), deacetylases (HDACs), methyltransferases (HMTs), and kinases (HKs), all acting directly on structural chromatin components. While others, including DNA methyltransferase enzymes (DNMTs), ten-eleven translocation proteins (TETs), and Thymine DNA glycosylase (TDG), are involved in the active DNA methylation/demethylation process. All these enzymes are responsible for the establishment of specific patterns that generate affinity for chromatin-associated proteins leading to their synergistic or antagonistic interaction, and resulting in the dynamic transitions between transcriptionally active or silent chromatin states, contributing to the cellular developmental plasticity and in particular contexts, leading to pathological outcomes [33, 40]. Moreover, ncRNAs—the transcribed part of the genome that lacks protein-coding potential [41]—play a significant role in post-transcriptional and gene expression regulation by establishing or mediating the epigenetic processes, for instance, by silencing or activating genes/transcripts through different mechanisms of action. ncRNAs can modulate cell behavior and have also been implicated as inter-cellular messengers [42]. Of note, depending on the subcellular localization, lncRNAs can mimic transcription factor binding sites, acting as a decoy, or can bind to exon/intron junctions of pre-mRNA and influence the splicing process [43]. Recently, researchers provide evidence about modifications in RNA molecules such as the methylation of adenine in position 1 (N1-methyladenosine, m1A) and 6 (N6-methyladenosine, m6A) performed by the N6-adenosine methyltransferase-like 3 (METTL3) [44–46]. In particular, mRNAs stability is altered when m6A occurs at their 5′-AGG (m6) AC-3′ consensus sequence, affecting their translation efficiency. Moreover, as for 5-methylcytosine (5mC), the m6A of RNA can be oxidized into N6-hydroximethyladenosine (hm6A) and N6-formyladenosine (f6A) and therefore demethylated, which might lead to a different RNA–protein interaction altering gene regulation. At the RNA level, these processes are catalyzed by the fat mass and obesity-associated protein (FTO) [47]. Noteworthy, RNA and DNA methyltransferases, such as members of the NOP2/Sun domain family and the DNA methyltransferase type 2 (DNMT2), respectively, can methylate ribocytidines at position 5 (5mC) [48, 49]. Interestingly, TET enzymes act similarly on DNA and RNA molecules, converting RNA 5mC into 5-hydroxymethylcytosine (5hmC), facilitating the translation of RNA molecules [50]. Likewise, methyl groups can also be transferred at position 7 of the riboguanosine (7-methylguanosine; m7G) [51]. This modification usually occurs on capped and recapped mRNAs and is mediated by canonical mRNA capping methyltransferase (RNMT), regulating mRNA translation into proteins [52]. Of note, thanks to the modern next-generation sequencing techniques, it was possible to create high-resolution epigenome maps of healthy and diseased cells, enabling simultaneous analysis of genetic and epigenetic changes, genome-wide genetic association studies (GWAS), and epigenome-wide association studies (EWAS), respectively [53].

## Epigenetic landscape alteration by viral infection

Tracking the epigenetic changes in pathophysiological contexts might represent an exciting source of knowledge to develop novel treatments leading, for example, to the regulation of the host immune response [54]. Most of the viruses belonging to the family of corona and influenza virus are usually incapable of hacking the host genetic sequence, while they might alter the host epigenome. Recent research has focused on how viruses utilize aspects of the epigenetic machinery to enable the infection establishment, spread, and persistence [55]. Furthermore, thanks to the recent advancements in high throughput technology, it is now possible to evaluate the epigenetic landscape at a genome-wide scale. It has been demonstrated that several viruses' families antagonize the immune system by employing a series of epigenetic mechanisms, and, likely, the SARS CoV-2 might use the same strategy.

Besides, several reports highlighted how viruses might disrupt the epigenetic network regulation impacting on the host immune response. For example, Marazzi et al. have shown how the highly pathogenic H3N2 influenza A virus inhibits the initiation of the host innate immune response by interfering with the epigenetic control of gene expression [56]. Exploiting histone mimicry, it has been demonstrated that the carboxy-terminus of the H3N2 nonstructural protein NS1 shares homolog sequences with the amino-terminus of the histone H3 tail [56]. Briefly, the viral NS1 protein mimics the histone tail of the H3 histone, interacting with the transcription complex, which usually docks to the H3K4 mark to initiate transcription, interfering with the antiviral gene function.

Similarly, both the hepatitis C virus (HCV) and adenoviruses have proteins that interfere with epigenetic functions and alter global immune function [57, 58]. In 2014, Baric's group found a clear association of repressive histone modification Histone 3-Lysine 27 trimethylation (H3K27me3) with down-regulated interferon (IFN)-stimulated genes (ISGs) following both MERS-CoV and influenza viruses A/influenza/Vietnam/1203/2004 (H5N1-VN1203) infection [59]; consequently, despite transcription factors and signaling pathways activation, the repressed state physically prevents transcription of these genes. More recently, Menachery and coworkers also observed that DNA methylation plays a similar role in the loss of antigen-presentation molecules following MERS-CoV and H5N1-VN1203 infection. Likewise, histone methylation was found involved in abating the immune response in H5N1, through the activity of the viral protein NS1 [60]. Importantly, their sequencing data suggested that other specific regions of the genome, perhaps encoding critical genes involved in viral antagonism, are also targeted by methylation. Specifically, DNA methylation was the primary suspect in suppressing the production of antigen presentation molecules in both diseases [60].

Another study by Schäfer and Baric indicated that SARS-CoV and MERS-CoV could delay or offset pathogen recognition and ISGs expression levels by encoding unique proteins that prevent immune signaling response [18]. Based on how other viruses like human immunodeficiency virus 1 (HIV-1) and herpes modulate chromatin, they suggested that these newer viruses may also act similarly [61, 62]. Interferons are essential mediators of antiviral actions and initiators of pathogen-driven immune response by the inactivation of ISGs [63, 64], and many viruses might develop antagonistic mechanisms to overcome specific ISG effectors [65]. Indeed, during the infection, IFN and innate immune responses are extensively regulated by specific epigenetic marks, through the manipulation of the epigenetic enzyme activity and chromatin remodeling complexes formation. The epigenetic machinery is responsible not only for the priming and the memory of host responses but also for ensuring their operational control. Fang et al. correlated the levels of Histone 3-Lysine 9 dimethylation (H3K9me2) with IFN expression in vitro. H3K9me2 is a repressive histone mark that controls the DNA methylation and heterochromatin formation processes. Specifically, the H3K9me2 mark hinders acetylation by recruiting the heterochromatin protein 1 family [66]. However, in the above study, Fang et al. demonstrated that the overall levels of H3K9me2 mark in the promoter region of the type I interferon and the expression of ISGs inversely correlate dendritic cells, defining this histone modification as an IFN response important regulator [66, 67].

On the other hand, the Histone 3-Lysine 4 trimethylation (H3K4me3) mark, commonly present in active promoters, is often enriched in Toll-like receptors (TLRs) promoter regions. Kaikkonen and coworkers have recently demonstrated that 60 min after lipopolysaccharide (LPS) stimulation of macrophages and dendritic cells, the overall histone acetylation and the binding of polymerase II (Pol II) to specific promoters was increased, suggesting a specific epigenetic regulation of the innate immune response induction [68]. Schäfer et al., using ChIP-PCR approaches, could determine differential occupancy of histone marks at the promoters of ISG genes, showing that the promoter regions of ISG genes contained more histones with active marks of H3K4 monomethylation (H3K4me) than the repressive H3K27me3 mark, therefore favoring open chromatin and promoting active transcription and ISG expression during H1N1 and SARS-CoV infection [18]. Otherwise, in MERS-CoV infected cells, Menachery et al*.* observed an increase in the H3K27me3 levels and reduced H3K4me3 levels at the promoter region of several specific ISGs subsets, which were not upregulated. These findings indicated that these viruses had developed antagonistic mechanisms to target the IFN innate immune response [59].

RNA type viruses, such as SARS-CoV, also show strong associations with RNA modifications. For instance, N6-methyladenosine (m6A) and N6,2′-O-dimethyladenosine (m6Am) modifications (m6A/m) have been found to play essential roles in the viral life cycle. In particular, they can affect the structure and replication of the virus, the host innate immune response, and some innate sensing pathways. The m6A RNA methylation is the most abundant epitranscriptomic modification of eukaryotic mRNAs and has been detected on cellular and viral transcripts, regulating numerous biological processes, including viral infection [69, 70]. Imam and colleagues suggested that m6A and its associated machinery regulate the DNA virus hepatitis B (HBV) life cycle, finalized through an RNA intermediate, termed pregenomic RNA (pgRNA). These observations indicated that m6A regulates HBV gene expression and reverse transcription. Indeed, by silencing the methylases that introduce the m6A modification to the RNA, they observed an increase in the HBV protein expression levels, while the pgRNA reverse transcription seemed reduced [71]. They mapped the m6A site in the HBV RNA and found that a conserved m6A consensus motif situated in the epsilon stem loop structure is the site for m6A modification. This loop is located in the 3′ terminus of all HBV mRNAs and at both the 5′ and 3′ ends of the pgRNA. Immam et al. identified an m6A site in the 5′ epsilon stem loop of pgRNA by mutational analysis, revealing that m6A is required for efficient reverse transcription of pgRNA. Furthermore, their finding suggested that m6A methylation of the 3′ epsilon stem loop resulted in the HBV transcripts destabilization, indicating a double regulatory function of m6A for HBV RNA.

Whereas, Tan and coworkers provided evidence that m6A and m6Am of messenger RNA mediate diverse cellular functions by examining the viral and cellular m6A/m epitranscriptomes during Kaposi's sarcoma-associated herpesvirus (KSHV) latent and lytic infection. KSHV transcripts are characterized by a high level of m6A/m modifications established during latent and lytic replication, conserved during the infection of different cell types [72]. Tan et al*.* showed that during lytic replication, upon YTH N6-methyladenosine RNA binding protein 2 (YTHDF2) knockdown, KSHV RNA degradation is impaired. YTHDF2 binds to viral transcripts and differentially mediates their stability. Moreover, they observed that KSHV latent infection-induced 5′ untranslated region (UTR) hypomethylation and 3′ UTR hypermethylation might alter the host epitranscriptome affecting the oncogenic and epithelial-mesenchymal transition processes. At the same time, KSHV lytic replication induces a dynamic reprogramming of the viral epitranscriptome itself [72].

Finally, Marz's group observed a consistent 5mC methylation signature of coronavirus RNA. Specifically, analyzing 5mC content across various RNAs, they observed consistent methylation patterns in corresponding genomic positions of different RNAs, suggesting that the methylation of coronavirus RNAs is sequence-specific or controlled by RNA structural elements [73]. Table 1 summarizes the epigenetic implication in viral infection and their functional outcomes.

**Table 1:** Relevant epigenetic implication in viral infection

Epigenetic modification	Virus infection	Target	Functional outcome
Histone methylation	H3N2 influenza A	H3K4	Inhibition of the initiation of the host innate immune response [55]
	SARS-CoV	H3K4me	Promotion of active transcription and ISG expression [16]
		H3K4me3	
	H1N1	H3K4me	Block of antiviral gene function [16, 54]
	MERS-CoV	H3K27me3	Down-regulation/inactivation of ISGs [16, 57, 63] and development of antagonistic mechanism to target the IFN innate immune response [59]
		H3K4me3	
	HSV	–	Down-regulation/inactivation of ISGs [59, 60]
	H5N1-Vn1203	H3K27me3	Down-regulation of ISGs [16, 57]
	HIV-1	–	Down-regulation/inactivation of ISGs [59, 60]
Histone acetylation	Adenovirus (Ad) E1A	H3K9ac	Interference with epigenetic functions and global immune function [55]
		H3K27ac	
DNA methylation	SARS-CoV	–	Delay/offset of pathogen recognition and modulation of ISG expression levels [16]
	MERS-CoV	–	Loss of antigen-presentation molecules [58]
	HSV	–	Delay/offset of pathogen recognition and modulation of ISG expression levels [16]
	H5N1-Vn1203	–	Loss of antigen-presentation molecules [58]
	HIV-1	–	Delay/offset of pathogen recognition and modulation of ISG expression levels [16]
	HCV	–	Interference with global immune function [56]
RNA methylation	KSHV	m6A/m6Am	Mediation of the stability of the viral transcripts [70]
	SARS-CoV	5mC	Modulation of the structure and the viral replication [67, 68]
	HBV	m6A	Regulation of gene expression and reverse transcription; transcript destabilization [69]

## Epigenetic implication in SARS- CoV-2 infection and therapy

In recent years, epigenetics evolved quickly, giving us better knowledge about inheritability functions, memory mechanisms, and developmental biology. The studies into the human epigenome are becoming more relevant in oncology, immunology, and infectious diseases [74, 75]. Indeed, during the last decade, the epigenetic research provided evidence that DNA and RNA viruses developed functions that antagonize the regulatory machine of the host epigenome by altering the host metabolism and gene expression, setting up a permissive environment for virus replication and spread [76, 77]. Furthermore, there is much evidence indicating that age-related changes to the host epigenome might compromise immune cell composition and function, affecting viral defenses, including the adaptive immune response [10, 12]. Coronaviruses, such as MERS-CoV and SARS-CoV-1, are known to mediate epigenetic alterations by antagonizing host antigen presentation or activating interferon-response genes [59, 60]. Evaluating the DNA methylation age of immune cells and other blood cell types before, during, and after infection could help explain how the aged epigenome impacts disease severity and how the virus alters the aged epigenome [10]. The vulnerability of the elderly to SARS-CoV-2 may also have to do with the effect of the epigenome on viral entry [78]. This process is initiated on the cell surface by physical interaction between the viral spike glycoprotein receptor, the ACE2 protein [26], and a co-receptor, the dipeptidyl peptidase-4 (DPP4) [30].

Nowadays, there are no specific antiviral drugs against COVID-19 infection yet, and vaccines are still under development. Even so, many potential therapeutic approaches are under investigation, and more research is urgently needed to identify effective vaccines and safe drugs for treating COVID-19 infections in order to develop pre- and post-exposure treatments against the pathogen. Although the first aim would be generating SARS-CoV-2 S-based vaccines, with conserved epitopes, able to elicit broadly neutralizing antibodies or virus-specific T cell responses, the identification, and development of safe and effective drugs to overcome SARS-CoV-2 entry and replication is essential.

Many strategies for COVID-19 treatment have been and still are under investigation: several antiviral drugs, among them Favipiravir (ClinicalTrials.gov Identifier: NCT04336904), Umifenovir (CTI: NCT04476719) or Lopinavir/Ritonavir (CTI: NCT04386876), alone or in combination with other chemicals such as the antimalarial chloroquine/hydroxychloroquine (e.g., CTI: NCT04328285); biologicals, such as convalescent plasma (e.g., CTI: NCT04321421) or mesenchymal stem cell (MSC) and MSC-derived exosomes (CTI: NCT04276987); Chinese traditional medicines (e.g., CTI: NCT04544605) and supplementation with Vitamins C and D (CTI: NCT03680274 and NCT04449718; https://www.clinicaltrials.gov/) [79–82]. Epigenetic research might help to accomplish these tasks thanks to a better understanding of the mechanisms involved in viral chromatin modification in lytic viruses and about host-virus interactions, including genetic factors that contribute to the protective or pathogenic host responses. Clinical trials, FDA approved epigenetic-targeted agents, and combination therapy of epigenetic and antiviral drugs is currently considered as useful and beneficial for viral replication impairment and the control of the host immune response [83]. Remarkably, pharmacokinetic and pharmacodynamic properties of antivirals may also be influenced by epigenetic regulation, highlighting, once again, their relevance in the treatment SARS-CoV-2 infection [84]. Recently, El Baba and coworkers analyzed several epigenetic mechanisms involved in coronaviruses infections, identifying some major epigenetic player which can be therapeutically targeted [83]. Indeed, many of the nonstructural proteins involved in viral transcription, replication, and maturation processes are regulated by different classes of HDACs, implying that HDAC inhibitors, such as Vorinostat or suberanilohydroxamic acid (SAHA), combined with antivirals, might be useful tools to interfere with these processes [85, 86]. Of note, previous studies already showed that ACE2 expression is regulated by DNA methylation and histone modifications. In this context, epigenetic enzymes responsible for the modifications mentioned above, such as DNMT1, histone acetyltransferase 1 (HAT1), histone deacetylase 2 (HDAC2), and lysine demethylase 5B (KDM5B), become potential targets to control the host immune response [87, 88]. Therefore, DNMT1 inhibitors, e.g., Azacitidine, HAT1 inhibitors, as the anacardic acid, and HDAC2 inhibitors, as the valproic acid, may be repurposed against CoVs infections [79, 82, 89].

Moreover, knowing that viruses depend on the host epigenetic machinery, epigenetic drugs already used in cancer therapies might be exploited for their broad-spectrum antiviral action and inflammatory control [83, 90]. Indeed, some evidence indicated that the main culprit behind COVID-19 deaths is the cytokine storm, characterized by an uncontrolled over-production of soluble markers of inflammation. Decitabine or 5-aza-2-deoxycytidine (5-azadC), a nucleoside-based DNMT inhibitor, is widely used to inhibit DNA methylation in macrophages; thus, suppressing inflammation and IFN response [83]. Noteworthy, Decitabine has recently been included in a clinical trial for COVID-19 Pneumonia-ARDS Treatment (CTI: NCT04482621).

Interestingly, the polycomb repressive complex 2 (PRC2), which mediates transcription repression via H3K27me3 enrichment at specific IFN-stimulated genes, could also be considered a target. Pharmacologic inhibitors of PRC2 are currently in advanced clinical trials for cancer treatment and could be easily repurposed to treat COVID-19 patients [91].

Recent studies show that innate immune cells may possess a form of memory, termed Trained Immunity (TRIM), a long-term boosting of innate immune response mainly maintained by natural killer cells and lung innate lymphoid cell group 2 through common epigenetic mechanisms [92, 93]. The exposure to an initial stimulus leads these cells to a metabolic, mitochondrial, and epigenetic reprogramming, which results in a memory phenotype of enhanced immune responses after the exposition to a secondary, heterologous stimulus [94]. Geller et al. also evaluate the potential effects of β-glucan about the immune dysregulation and cytokine storm observed in COVID-19. In their studies, they observed that β-glucan-driven TRIM also determines some epigenetic changes and that it could represent a useful target for COVID-19 treatment [94].

Recent studies also propose vitamins and natural products, as epigenetic modifiers, to enhance immunity and reduce the inflammatory response in COVID-19 patients [95–97]. For instance, the use of Vitamin D and quercetin could be interesting for ameliorating SARS-Cov-2 severity by inhibiting the expression of ACE2 and its possible role in suppressing the cytokine storm associated with mortality in COVID-19 patients [96, 98].

**Fig. 2:**
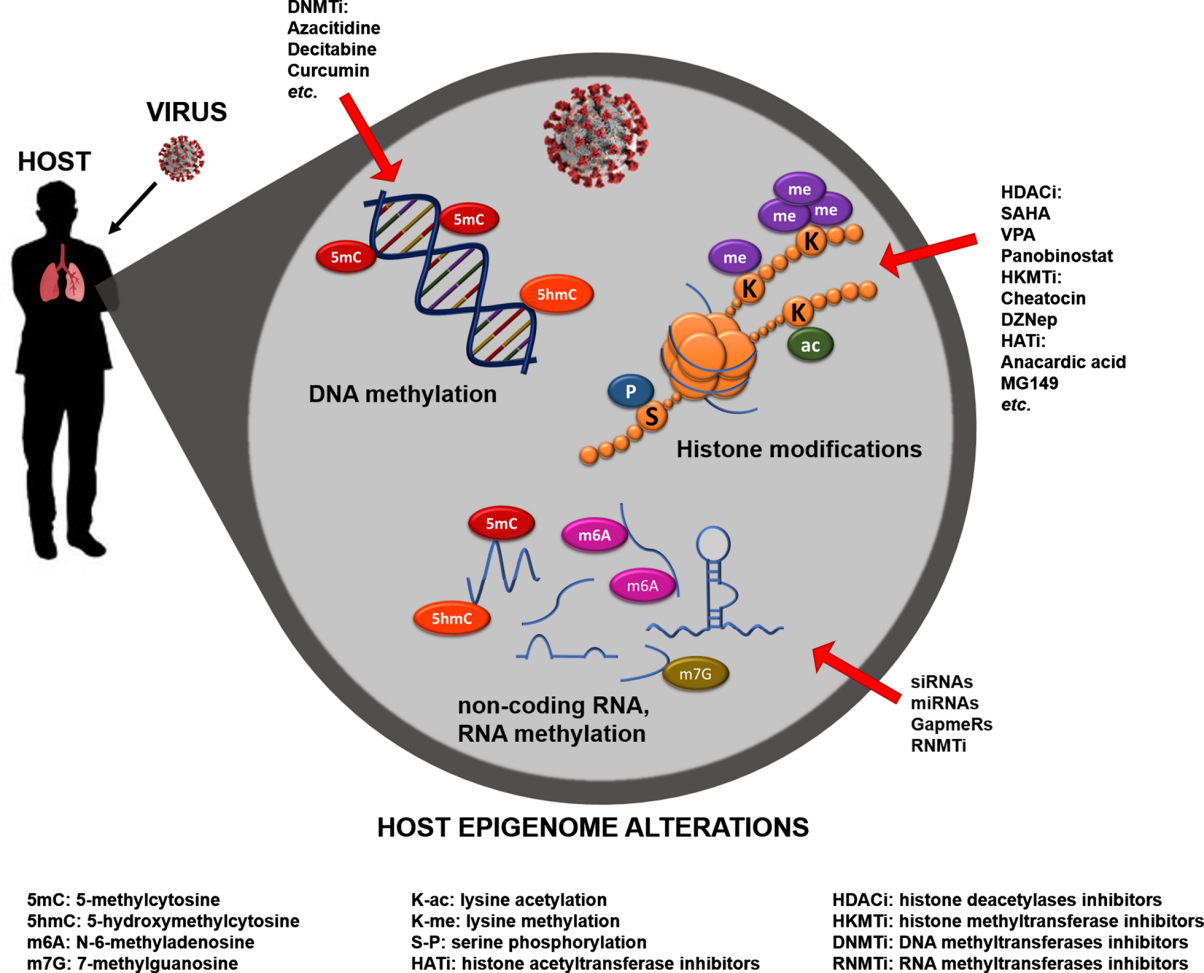
Coronavirus-dependent host epigenome alterations and potential interventions. Viruses, like those from the Coronaviridae family, can alter the host epigenome, negatively affecting the host immune response and successfully spreading the infection. The immune response is extensively regulated by specific epigenetic marks, such as chromatin remodeling, histone modification, DNA, and RNA methylation. The epigenetic machinery is responsible not only for the host response priming and memory, but also for ensuring its functional regulation. Age-related alterations to the host epigenome might affect the adaptive immune response, hindering viral defenses. Epigenomics represent a powerful tool to explore how to prevent, attenuate, or reverse the viral infection therapeutically. The enzymes responsible for the epigenetic alterations might represent potential targets for new antiviral drugs

RNA-based drugs are other epigenetic tools that should be investigated for treating viral infections [99, 100]. For instance, among all the SARS-CoV genome that have been under study so far, Baldassarre and coworkers suggested that the 5′URT region and specific portion of it, which are essential for viral RNA replication and transcription, could be considered relevant to design novel therapeutic molecules to treat the infection [101]. Novel strategies employing small interfering RNAs (siRNAs), microRNAs (miRNAs), and locked nucleic acid antisense oligonucleotides (LNA) or GapmeRs, targeting, for instance, the 5′URT or regions of the Spike molecule, represent potential therapeutic tools for both prophylaxis and therapy of COVID-19 [101–103]. Indeed, the design of antisense oligonucleotides, such as Miravirsen, under investigation for HCV treatment, could be used to inhibit viral replication by scavenging miRNAs that are involved in the process [104, 105]. These studies suggest that RNA-based drugs could be optimized and employed to interfere with SARS-CoV-2 replication and transcription. Figure 2 summarizes some of the epigenetic targets and interventions potentially useful for Coronavirus viral infections treatment.

Noteworthy, thanks to sophisticated bioinformatics software, we are now able to visualize and interpret the epigenomic data, providing in-depth cell-specific knowledge about the genetic and epigenetic predispositions of an individual and explaining how the environment affects the function of our genes by leaving long-term marks on the genome. Indeed, epigenome mapping, together with EWAS and GWAS studies, provides us with tools in the diagnostics of many common human diseases, indicating that these studies could be employed for individual diagnosis and personalized therapies [106, 107]. Therefore, by investigating the epigenetic effects of metabolism from genes to pathways to genomes, and thanks to the availability of the novel detailed epigenomic datasets, we may explore how to therapeutically prevent, attenuate, or reverse the epigenetic alterations, how to design and realize specific pharmacological tools and when/where to intervene. Above all, the enzymes responsible for the epigenetic alterations represent an exciting field for discovering new drug targets.

## Conclusions

The COVID-19 pandemic is one of the most serious global health threats of the contemporary age so far. The presence of the so-called cytokine storm induced by the virus leads to ARDS aggravation and widespread tissue damage resulting in multi-organ failure and death. SARS-CoV-2 infection, by interfering with the host epigenetic machinery, might alter the expression of proinflammatory cytokines, such as IL-1, IL-6, IL-18, IFN-γ, and TNF-α. Epigenomic studies might open new avenues for developing antiviral drugs by evaluating specific epigenetic modulators as targets and exploring new chromatin-based therapies for different virus families, including Coronaviruses, which could reveal fundamental new landscapes of virus–host interaction and their role in disease severity [85]. Previous works focused on specific epigenetic mechanisms [83, 101]. This article summarizes the comprehensive knowledge about epigenetic aspects associated with SARS-CoV-2 infection and suggests potential epigenetically based therapies. In particular, from this analysis, it emerges that understanding the epigenetic regulation underlying the immune response to SARS-CoV-2 will help to design and develop novel specific strategies to prevent and treat the infection.

## Data Availability

Not applicable.

## Abbreviations

5mC: 5-Methylcytosine
5hmC: 5-Hydroxymethylcytosine
ACE2: Angiotensin-converting enzyme 2
ARD: Age-related diseases
ARDS: Severe symptomatic acute respiratory distress
COVID-19: Coronavirus disease 19
DNMT: DNA methyltransferases
DNMT2: DNA methyltransferase type 2
DPP4: Dipeptidyl peptidase 4
E: Envelope protein
ERGIC: Endoplasmic reticulum-Golgi intermediate complex
EWAS: Epigenome-wide association studies
f6A: N6-formyladenosine
FTO: Fat mass and obesity-associated protein
GWAS: Genome-wide genetic association studies
H3: Histone 3
H3K27me3: Histone 3-Lysine 27 trimethylation
H3K4me: Histone 3-Lysine 4 monomethylation
H3K4me3: Histone 3-Lysine 4 trimethylation
H3K9me2: Histone 3-Lysine 9 dimethylation
H5N1-VN1203: Influenza viruses A/influenza/Vietnam/1203/2004
HATs: Histone acetyltransferases
HBV: Hepatitis B virus
HCV: Hepatitis C virus
HDACs: Histone deacetylases
HIV-1: Human immunodeficiency virus 1
HKs: Histone kinases
hm6A: N6-hydroximethyladenosine
HMT: Histone methyltransferases
IFN: Interferon
ISGs: Interferon-stimulated genes
KSHV: Kaposi's sarcoma-associated herpesvirus
LNA: Locked nucleic acid antisense oligonucleotides
LPS: Lipopolysaccharide
M: Membrane protein
m1A: N1-Methyladenosine
m6A: N6-Methyladenosine
m6Am: N6,2′-O-Dimethyladenosine
m6A/m: N6-Methyladenosine and N6,2′-O-Dimethyladenosine modifications
m7G: 7-Methylguanosine
MERS-CoV: Middle East respiratory syndrome
METTL3: N6-adenosine methyltransferase-like 3
miRNAs: MicroRNAs
Nsp: Nonstructural proteins
N: Nucleocapsid protein
ORFs: Open‐reading frames
pgRNA: Pregenomic RNA
Pol II: Polymerase II
RNMT: mRNA capping methyltransferase
S: Spike protein
SARS-CoV: Severe acute respiratory syndrome
siRNAs: Small interfering RNAs
TDG: Thymine DNA glycosylase
TETs: Ten-eleven translocation proteins
TLRs: Toll-like receptors
TRIM: Trained immunity
UTR: Untranslated region
YTHDF2: YTH N6-methyladenosine RNA binding protein 2

## References

[1] Zhou P, Yang X-L, Wang XG, Hu B, Zhang L, Zhang W (2020). A pneumonia outbreak associated with a new coronavirus of probable bat origin. Nature.

[2] Whitworth J (2020). COVID-19: a fast evolving pandemic. Trans R Soc Trop Med Hyg.

[3] Rampal L, Liew BS (2020). Coronavirus disease (COVID-19) spreads situation reports. WHO..

[4] Qing E, Gallagher T (2020). SARS coronavirus redux. Trends Immunol.

[5] Zhu N, Zhang D, Wang W, Li X, Yang B, Song J (2020). A novel coronavirus from patients with pneumonia in China, 2019. N Engl J Med.

[6] Benvenuto D, Giovanetti M, Ciccozzi A, Spoto S, Angeletti S, Ciccozzi M (2020). The 2019-new coronavirus epidemic: evidence for virus evolution. J Med Virol.

[7] Lu R, Zhao X, Li J, Niu P, Yang B, Wu H (2020). Genomic characterisation and epidemiology of 2019 novel coronavirus: implications for virus origins and receptor binding. Lancet.

[8] Guan WJ, Liang WH, Zhao Y, Liang HR, Chen ZS, Li YM (2020). Comorbidity and its impact on 1,590 patients with Covid-19 in China: a nationwide analysis. Eur Respir J.

[9] Li B, Yang J, Zhao F, Zhi L, Wang X, Liu L (2020). Prevalence and impact of cardiovascular metabolic diseases on COVID-19 in China. Clin Res Cardiol.

[10] Mueller AL, Mcnamara MS, Sinclair DA (2020). Why does COVID-19 disproportionately affect older people?. Aging (Albany NY).

[11] López-Otín C, Blasco MA, Partridge L, Serrano M, Kroemer G (2013). The hallmarks of aging. Cell.

[12] Salimi S, Hamlyn JM (2020). COVID-19 and crosstalk with the hallmarks of aging. J Gerontol A Biol Sci Med Sci.

[13] Lu L, Zhong W, Bian Z, Li Z, Zhang K, Liang B (2020). A comparison of mortality-related risk factors of COVID-19, SARS, and MERS: a systematic review and meta-analysis. J Infect.

[14] Mori H, Obinata H, Murakami W, Tatsuya K, Sasaki H, Miyake Y (2020). Comparison of COVID-19 disease between young and elderly patients: hidden viral shedding of COVID-19. J Infect Chemother.

[15] Shereen MA, Khan S, Kazmi A, Bashir N, Siddique R (2020). COVID-19 infection: origin, transmission, and characteristics of human coronaviruses. J Adv Res.

[16] Chen Y, Liu Q, Guo D (2020). Emerging coronaviruses: genome structure, replication, and pathogenesis. J Med Virol.

[17] Kandeel M, Ibrahim A, Fayez M, Al-Nazawi M (2020). From SARS and MERS CoVs to SARS-CoV-2: moving toward more biased codon usage in viral structural and nonstructural genes. J Med Virol.

[18] Schäfer A, Baric RS (2017). Epigenetic landscape during coronavirus infection. Pathogens.

[19] Hulswit RJG, de Haan CAM, Bosch B-J (2016). Coronavirus spike protein and tropism changes. Adv Virus Res.

[20] Risco C, Antón IM, Enjuanes L, Carrascosa JL (1996). The transmissible gastroenteritis coronavirus contains a spherical core shell consisting of M and N proteins. J Virol.

[21] Ruch TR, Machamer CE (2012). The coronavirus E protein: assembly and beyond. Viruses.

[22] Neuman BW, Kiss G, Kunding AH, Bhella D, Baksh MF, Connelly S (2011). A structural analysis of M protein in coronavirus assembly and morphology. J Struct Biol.

[23] Wang Q, Zhang Y, Wu L, Niu S, Song C, Zhang Z (2020). Structural and functional basis of SARS-CoV-2 entry by using human ACE2. Cell.

[24] Letko M, Marzi A, Munster V (2020). Functional assessment of cell entry and receptor usage for SARS-CoV-2 and other lineage B betacoronaviruses. Nat Microbiol.

[25] Yan R, Zhang Y, Li Y, Xia L, Guo Y, Zhou Q (2020). Structural basis for the recognition of SARS-CoV-2 by full-length human ACE2. Science.

[26] Hoffmann M, Kleine-Weber H, Schroeder S, Krüger N, Herrler T, Erichsen S (2020). SARS-CoV-2 cell entry depends on ACE2 and TMPRSS2 and is blocked by a clinically proven protease inhibitor. Cell.

[27] Li W, Zhang C, Sui J, Kuhn JH, Moore MJ, Luo S (2005). Receptor and viral determinants of SARS-coronavirus adaptation to human ACE2. EMBO J.

[28] Park YJ, Walls AC, Wang Z, Sauer MM, Li W, Tortorici MA (2019). Structures of MERS-CoV spike glycoprotein in complex with sialoside attachment receptors. Nat Struct Mol Biol.

[29] Kim KM, Noh JH, Bodogai M, Martindale JL, Yang X, Indig FE (2017). Identification of senescent cell surface targetable protein DPP4. Genes Dev.

[30] Vankadari N, Wilce JA (2020). Emerging WuHan (COVID-19) coronavirus: glycan shield and structure prediction of spike glycoprotein and its interaction with human CD26. Emerg Microbes Infect.

[31] Strollo R, Pozzilli P (2020). DPP4 inhibition: preventing SARS-CoV-2 infection and/or progression of COVID-19?. Diabetes Metab Res Rev.

[32] Berger SL, Kouzarides T, Shiekhattar R, Shilatifard A (2009). An operational definition of epigenetics. Genes Dev.

[33] Jin Z, Liu Y (2018). DNA methylation in human diseases. Genes Dis.

[34] Goldberg AD, Allis CD, Bernstein E (2007). Epigenetics: a landscape takes shape. Cell.

[35] Keating ST, El-Osta A (2015). Epigenetics and metabolism. Circ Res.

[36] Jaenisch R, Bird A (2003). Epigenetic regulation of gene expression: How the genome integrates intrinsic and environmental signals. Nat Genet.

[37] Wu H, Zhang Y (2014). Reversing DNA methylation: mechanisms, genomics, and biological functions. Cell.

[38] Dunham I, Kundaje A, Aldred SF, Collins PJ, Davis CA, Doyle F (2012). An integrated encyclopedia of DNA elements in the human genome. Nature.

[39] Luger K, Mäder AW, Richmond RK, Sargent DF, Richmond TJ (1997). Crystal structure of the nucleosome core particle at 2.8 Å resolution. Nature.

[40] Bannister AJ, Kouzarides T (2011). Regulation of chromatin by histone modifications. Cell Res.

[41] Beermann J, Piccoli M-T, Viereck J, Thum T (2016). Non-coding RNAs in development and disease: background, mechanisms, and therapeutic approaches. Physiol Rev.

[42] De Majo F, Calore M (2018). Chromatin remodelling and epigenetic state regulation by non-coding RNAs in the diseased heart. Non-coding RNA Res.

[43] Boon RA, Jaé N, Holdt L, Dimmeler S (2016). Long noncoding RNAs from clinical genetics to therapeutic targets?. J Am Coll Cardiol.

[44] Xiong X, Li X, Yi C (2018). N1-methyladenosine methylome in messenger RNA and non-coding RNA. Curr Opin Chem Biol.

[45] Liu J, Eckert MA, Harada BT, Liu SM, Lu Z, Yu K (2018). m 6 A mRNA methylation regulates AKT activity to promote the proliferation and tumorigenicity of endometrial cancer. Nat Cell Biol.

[46] Mongelli A, Atlante S, Bachetti T, Martelli F, Farsetti A, Gaetano C (2020). Epigenetic signaling and RNA regulation in cardiovascular diseases. Int J Mol Sci..

[47] Fu Y, Jia G, Pang X, Wang RN, Wang X, Li CJ (2013). FTO-mediated formation of N6-hydroxymethyladenosine and N 6-formyladenosine in mammalian RNA. Nat Commun.

[48] Jeltsch A, Ehrenhofer-Murray A, Jurkowski TP, Lyko F, Reuter G, Ankri S (2017). Mechanism and biological role of Dnmt2 in nucleic acid methylation. RNA Biol.

[49] Brzezicha B, Schmidt M, Makałowska I, Jarmołowski A, Pieńkowska J, Szweykowska-Kulińska Z (2006). Identification of human tRNA: m5C methyltransferase catalysing intron-dependent m5C formation in the first position of the anticodon of the pre-tRNA(CAA)Leu. Nucleic Acids Res.

[50] Delatte B, Wang F, Ngoc LV, Collignon E, Bonvin E, Deplus R (2016). Transcriptome-wide distribution and function of RNA hydroxymethylcytosine. Science.

[51] Leulliot N, Chaillet M, Durand D, Ulryck N, Blondeau K, van Tilbeurgh H (2008). Structure of the yeast tRNA m7G methylation complex. Structure.

[52] Trotman JB, Giltmier AJ, Mukherjee C, Schoenberg DR (2017). RNA guanine-7 methyltransferase catalyzes the methylation of cytoplasmically recapped RNAs. Nucleic Acids Res.

[53] Paul DS, Beck S (2014). Advances in epigenome-wide association studies for common diseases. Trends Mol Med.

[54] Smale ST, Tarakhovsky A, Natoli G (2014). Chromatin contributions to the regulation of innate immunity. Annu Rev Immunol.

[55] Lieberman PM (2016). Epigenetics and genetics of viral latency. Cell Host Microbe.

[56] Marazzi I, Garcia-Sastre A (2015). Interference of viral effector proteins with chromatin, transcription, and the epigenome. Curr Opin Microbiol.

[57] Ferrari R, Gou D, Jawdekar G, Johnson SA, Nava M, Su T (2014). Adenovirus small E1A employs the lysine acetylases p300/CBP and tumor suppressor RB to repress select host genes and promote productive virus infection. Cell Host Microbe.

[58] Seo YL, Heo S, Jang KL (2015). Hepatitis C virus core protein overcomes H2O2-induced apoptosis by downregulating p14 expression via DNA methylation. J Gen Virol.

[59] Menachery VD, Eisfeld AJ, Schäfer A, Josset L, Sims AC, Proll S (2014). Pathogenic influenza viruses and coronaviruses utilize similar and contrasting approaches to control interferon-stimulated gene responses. MBio.

[60] Menachery VD, Schäfer A, Burnum-Johnson KE, Mitchell HD, Eisfeld AJ, Walters KB (2018). MERS-CoV and H5N1 influenza virus antagonize antigen presentation by altering the epigenetic landscape. Proc Natl Acad Sci U S A.

[61] Van Lint C, Emiliani S, Ott M, Verdin E (1997). Transcriptional activation and chromatin remodeling of the HIV-I promoter in response to histone acetylation. Chemtracts.

[62] Liang Y, Vogel JL, Narayanan A, Peng H, Kristie TM (2009). Inhibition of the histone demethylase LSD1 blocks α-herpesvirus lytic replication and reactivation from latency. Nat Med.

[63] Ivashkiv LB, Donlin LT (2014). Regulation of type i interferon responses. Nat Rev Immunol.

[64] Schneider WM, Chevillotte MD, Rice CM (2014). Interferon-stimulated genes: a complex web of host defenses. Annu Rev Immunol.

[65] García-Sastre A, Biron CA (2006). Type 1 interferons and the virus-host relationship: a lesson in détente. Science.

[66] Fang TC, Schaefer U, Mecklenbrauker I, Stienen A, Dewell S, Chen MS (2012). Histone H3 lysine 9 di-methylation as an epigenetic signature of the interferon response. J Exp Med.

[67] Aevermann BD, Pickett BE, Kumar S, Klem EB, Agnihothram S, Askovich PS (2014). A comprehensive collection of systems biology data characterizing the host response to viral infection. Sci Data.

[68] Kaikkonen MU, Lam MTY, Glass CK (2011). Non-coding RNAs as regulators of gene expression and epigenetics. Cardiovasc Res.

[69] Lichinchi G, Gao S, Saletore Y, Gonzalez GM, Bansal V, Wang Y (2016). Dynamics of the human and viral m(6)A RNA methylomes during HIV-1 infection of T cells. Nat Microbiol.

[70] Kennedy EM, Bogerd HP, Kornepati AVR, Kang D, Ghoshal D, Marshall JB (2016). Posttranscriptional m6A editing of HIV-1 mRNAs enhances viral gene expression. Cell Host Microbe.

[71] Imam H, Khan M, Gokhale NS, McIntyre ABR, Kim GW, Jang JY (2018). N6-methyladenosine modification of hepatitis b virus RNA differentially regulates the viral life cycle. Proc Natl Acad Sci U S A.

[72] Tan B, Gao SJ (2018). RNA epitranscriptomics: Regulation of infection of RNA and DNA viruses by N6-methyladenosine (m6A). Rev Med Virol.

[73] Viehweger A, Krautwurst S, Lamkiewicz K, Madhugiri R, Ziebuhr J, Hölzer M (2019). Direct RNA nanopore sequencing of full-length coronavirus genomes provides novel insights into structural variants and enables modification analysis. Genome Res.

[74] Portela A, Esteller M (2010). Epigenetic modifications and human disease. Nat Biotechnol.

[75] Obata Y, Furusawa Y, Hase K (2015). Epigenetic modifications of the immune system in health and disease. Immunol Cell Biol.

[76] Busslinger M, Tarakhovsky A (2014). Epigenetic control of immunity. Cold Spring Harb Perspect Biol.

[77] Vavougios GD (2020). A data-driven hypothesis on the epigenetic dysregulation of host metabolism by SARS coronaviral infection: potential implications for the SARS-CoV-2 modus operandi. Med Hypotheses.

[78] Holt N, Neumann J, McNeil J, Cheng A (2020). Implications of COVID-19 in an ageing population. Med J Aust.

[79] Gordon DE, Jang GM, Bouhaddou M, Xu J, Obernier K, White KM (2020). A SARS-CoV-2 protein interaction map reveals targets for drug repurposing. Nature.

[80] Zhang L, Liu Y (2020). Potential interventions for novel coronavirus in China: a systematic review. J Med Virol.

[81] Wang M, Cao R, Zhang L, Yang X, Liu J, Xu M (2020). Remdesivir and chloroquine effectively inhibit the recently emerged novel coronavirus (2019-nCoV) in vitro. Cell Res.

[82] Li G, De Clercq E (2020). Therapeutic options for the 2019 novel coronavirus (2019-nCoV). Nat Rev Drug Discov.

[83] El Baba R, Herbein G (2020). Management of epigenomic networks entailed in coronavirus infections and COVID-19. Clin Epigenet.

[84] Paniri A, Mahdi M, Rasoulinejad A (2020). Molecular effects and retinopathy induced by hydroxychloroquine during SARS-CoV-2 therapy: role of CYP450 isoforms and epigenetic modulations. Eur J Pharmacol.

[85] Nehme Z, Pasquereau S, Herbein G (2019). Control of viral infections by epigenetic-targeted therapy. Clin Epigenet.

[86] Cole J, Morris P, Dickman MJ, Dockrell DH (2016). The therapeutic potential of epigenetic manipulation during infectious diseases. Pharmacol Ther.

[87] Chlamydas S, Papavassiliou AG, Piperi C (2020). Epigenetic mechanisms regulating COVID-19 infection. Epigenetics.

[88] Chai P, Yu J, Ge S, Jia R, Fan X (2020). Genetic alteration, RNA expression, and DNA methylation profiling of coronavirus disease 2019 (COVID-19) receptor ACE2 in malignancies: a pan-cancer analysis. J Hematol Oncol.

[89] Dekker FJ, Van Den Bosch T, Martin NI (2014). Small molecule inhibitors of histone acetyltransferases and deacetylases are potential drugs for inflammatory diseases. Drug Discov Today.

[90] Van Dam PA, Huizing M, Mestach G, Dierckxsens S, Tjalma W, Trinh XB (2020). SARS-CoV-2 and cancer: are they really partners in crime?. Cancer Treat Rev.

[91] Ayaz S, Crea F (2020). Targeting SARS-CoV-2 using polycomb inhibitors as antiviral agents. Epigenomics.

[92] Netea MG, Giamarellos-Bourboulis EJ, Domínguez-Andrés J, Curtis N, van Crevel R, van de Veerdonk FL (2020). Trained immunity: a tool for reducing susceptibility to and the severity of SARS-CoV-2 infection. Cell.

[93] Kerboua KE (2020). The perplexing question of trained immunity versus adaptive memory in COVID-19. J Med Virol.

[94] Geller A, Yan J (2020). Could the induction of trained immunity by β-glucan serve as a defense against COVID-19?. Front Immunol.

[95] Singh V (2020). Can vitamins, as epigenetic modifiers, enhance immunity in COVID-19 patients with non-communicable disease?. Curr Nutr Rep.

[96] Vyas N, Kurian SJ, Bagchi D, Manu MK, Saravu K, Unnikrishnan MK (2020). Vitamin D in prevention and treatment of COVID-19: current perspective and future prospects. J Am Coll Nutr.

[97] Fang Y, Yang C, Yu Z, Li X, Mu Q, Liao G (2020). Natural products as LSD1 inhibitors for cancer therapy. Acta Pharm Sin B.

[98] Pruimboom L (2020). Methylation pathways and SARS-CoV-2 lung infiltration and cell membrane-virus fusion are both subject to epigenetics. Front Cell Infect Microbiol.

[99] Wu C-J, Chan Y-L (2006). Antiviral applications of RNAi for coronavirus. Expert Opin Investig Drugs.

[100] Levanova A, Poranen MM (2018). RNA interference as a prospective tool for the control of human viral infections. Front Microbiol.

[101] Baldassarre A, Paolini A, Bruno SP, Felli C, Tozzi AE, Masotti A (2020). Potential use of noncoding RNAs and innovative therapeutic strategies to target the 5’UTR of SARS-CoV-2. Epigenomics.

[102] Zhang Y, Li T, Fu L, Yu C, Li Y, Xu X (2004). Silencing SARS-CoV Spike protein expression in cultured cells by RNA interference. FEBS Lett.

[103] Zheng BJ, Guan Y, Tang O, Cheng D, Xie FY, He ML (2004). Prophylactic and therapeutic effects of small interfering RNA targeting SARS-coronavirus. Antivir Ther.

[104] Janssen HLA, Reesink HW, Lawitz EJ, Zeuzem S, Rodriguez-Torres M, Patel K (2013). Treatment of HCV infection by targeting microRNA. N Engl J Med.

[105] Verma NK, Fazil MHUT, Duggan SP, Kelleher D (2020). Combination therapy using inhalable GapmeR and recombinant ACE2 for COVID-19. Front Mol Biosci.

[106] Thornbrough JM, Jha BK, Yount B, Goldstein SA, Li Y, Elliott R (2016). Middle east respiratory syndrome coronavirus. Work Heal Saf.

[107] Rabouw HH, Langereis MA, Knaap RCM, Dalebout TJ, Canton J, Sola I (2016). Middle east respiratory coronavirus accessory protein 4a inhibits pkr-mediated antiviral stress responses. PLoS Pathog.

